# The Role of ETNPPL in Dopaminergic Neuron Stability: Insights from Neuromelanin-Associated Protein Expression in Parkinson’s Disease

**DOI:** 10.3390/ijms252313107

**Published:** 2024-12-06

**Authors:** Francesca A. Schillaci, Giuseppe Lanza, Maria Grazia Salluzzo, Francesca L’Episcopo, Raffaele Ferri, Michele Salemi

**Affiliations:** 1 Oasi Research Institute-IRCCS, 94018 Troina, Italy; fran7.sch@gmail.com (F.A.S.); glanza@oasi.en.it (G.L.); msalluzzo@oasi.en.it (M.G.S.); flepiscopo@oasi.en.it (F.L.); msalemi@oasi.en.it (M.S.); 2 Department of Surgery and Medical-Surgical Specialties, University of Catania, 95125 Catania, Italy

**Keywords:** Parkinson’s disease, immunohistochemistry, qRT-PCR, dopaminergic neurons, substantia nigra, ETNPPL protein

## Abstract

More than six million people worldwide are affected by Parkinson’s disease (PD), a multifactorial disorder characterized by the progressive loss of dopaminergic neurons in the substantia nigra pars compacta (SNc). Several immunohistochemical studies suggest that neuromelanin (NM), found in these neurons, plays a key role in their degeneration. In this study, twelve formalin-fixed, paraffin-embedded (FFPE) brain sections were analyzed, comprising six samples from PD patients and six from healthy controls. Immunohistochemistry (IHC) was conducted to assess the expression of the ETNPPL protein in these samples. ETNPPL was detected in both PD and control samples. Additionally, we examined the expression of ETNPPL mRNA using Quantitative Real-Time PCR (qRT-PCR) in the same sample set. Notably, in control samples, ETNPPL protein was closely associated with the dark NM pigment in the cytoplasm of SNc dopaminergic neurons. In contrast, PD samples showed weak cytoplasmic expression of ETNPPL, with no association with the NM pigment. No nuclear ETNPPL signal was detected in dopaminergic neurons from either PD patients or controls. qRT-PCR results revealed lower ETNPPL mRNA expression in individual PD patients compared to controls. Importantly, we observed a higher concentration of ETNPPL protein at the NM level in the SNc neurons of controls, consistent with mRNA expression patterns. These findings suggest a potential role for ETNPPL in the normal function of dopaminergic neurons and underscore its altered expression in Parkinson’s disease.

## 1. Introduction

More than six million people are estimated to suffer from Parkinson’s disease (PD) worldwide, with a 2.5-fold increase in prevalence compared to the previous generation, as reported by the Global Burden of Disease. Along with Alzheimer’s dementia, PD is considered a leading cause of neurological disability, particularly in the elderly [1,2]. However, PD is highly heterogeneous in terms of age of onset (ranging between 45 and 80 years), clinical presentation, rate of disease progression, and response to treatment, which varies from one individual to another [1]. Pathogenically, PD is a multifactorial disorder, primarily resulting from a combination of genetic and environmental factors that often act synergistically. Despite advancements in early and accurate diagnosis [3], understanding the interactions at an individual level between causal and protective factors—referred to as the “environmentome”—is not yet possible [4,5], limiting both prevention strategies and therapeutic options.

PD is typically divided into three stages: preclinical PD, premotor or prodromal PD, and clinical PD. In the clinical stage, motor signs confirm the presence of overt disease, including bradykinesia, muscle rigidity, resting tremor, and, in more advanced stages, postural changes and gait disturbances. These motor symptoms are linked to nigral degeneration and α-synuclein-mediated striatal dopamine depletion. In the prodromal phase, certain non-motor signs—such as hyposmia, constipation, REM sleep behavior disorder, and depression—are thought to result from the degeneration of other structures, including the peripheral autonomic nervous system and the brainstem [1,6,7,8].

The pathophysiology of PD appears to be more complex than clinically anticipated. According to the primary hypothesis, it involves a combination of factors such as abnormal alpha-synuclein aggregation, mitochondrial dysfunction, lysosomal or vesicle transport impairments, synaptic dysfunction, microRNA dysregulation, and neuroinflammatory processes. These factors contribute to the progressive loss of dopaminergic neurons in the substantia nigra pars compacta (SNc) due to the destruction of pigmented neurons (which contain neuromelanin, or NM) and the presence of pathological neuronal inclusions (Lewy bodies) in the SNc and other brain regions. These pathological changes can accumulate for years, even decades, before clinical symptoms emerge (preclinical PD) [1,4,5,9,10,11,12].

The impact of PD on the SNc has been well-established since 1953. There is a significant loss of pigmented neurons in this area, leading to a reduction in dopamine; however, the physiological and/or pathological significance of NM remains unclear [12,13,14]. NM was first described by Purkinje in 1838 [15]. Histochemical studies have shown that NM is not only located in the SNc but also in the locus coeruleus and shares melanin-like characteristics, such as insolubility in organic solvents, bleaching by hydrogen peroxide, and staining with silver [12,16]. It was named ‘neuromelanin’ due to its location in the brain, and the term “substantia nigra” was derived from its melanin content, which gives it a dark brown pigment [13,14,16].

Several immunohistochemical studies have demonstrated significant depletion of nigral neurons in individuals with PD. Kurosaki et al. [17] conducted a study on mice in which parkinsonism was induced by 1-methyl-4-phenyl-1,2,3,6-tetrahydropyridine hydrochloride (MPTP). They concluded that nigral degeneration leads to motor abnormalities. Their immunohistochemical analysis suggested that an impaired ability to quench free radicals may play a key role in neuronal damage following MPTP neurotoxicity. Yoritaka’s [18] immunohistochemical study focused on mitochondrial dysfunction, specifically oxidative stress and mitochondrial respiratory failure, which were observed in individuals with PD and contributed to nigral neuron death. Using histochemistry, they identified PD-related oxidative damage sites by applying an antiserum against modified 4-hydroxy-2-nonenal (HNE) protein in both PD patients and healthy controls. They deduced that increased lipid peroxidation in nigral neurons may seriously impair membrane functions such as oxidative phosphorylation, signal transduction, and the regulation of electron, iron, and metabolite transport, contributing to PD neurodegeneration [18].

Immunohistochemistry (IHC) is a widely used technique in both diagnostics and research, particularly for neurodegenerative diseases. It allows for the detection of cellular or tissue antigens—such as amino acids, proteins, infectious agents, and other cell populations—by visualizing the formation of antigen–antibody (Ag-Ab) complexes in tissue sections from both animals and humans. This is made visible through histochemical staining observed via light microscopy or fluorochrome labeling under ultraviolet light [19].

In this complex context, we recently analyzed mRNAs extracted post-mortem from the SNc of PD patients and healthy controls [20]. Among the under-expressed mRNAs in PD compared to controls (padj ≤ 0.05 and |FC| ≥ 1.5), we identified the ethanolamine-phosphate-lyase gene (*ETNPPL*/*AGXT2L1*), which resulted to be at the top of the list of genes differentially expressed obtained from the previous work (Tables 1 and 2 from [20]). In addition, in the same study [20], we identified several molecular networks obtained by Ingenuity Pathway Analysis (IPA) which were differentially activated in PD compared to controls (Figures 2–4 and Table 3 from [20]). *ETNPPL* is involved in the catabolism of phosphoethanolamine (PEtN), a crucial compound for synthesizing phosphatidylethanolamine, a major phospholipid of cell membranes [21]. Veiga-da-Cunha et al. [22] identified the activity of AGXT2L1 (which is an alternative name of ETNPPL) and AGXT2L2, suggesting that the former may play an important function in the maintenance of cell membranes, metabolism of PEtN, and control of its concentration within the CNS; thus, its dysregulation might have a significant impact in neurological disorders.

Moreover, an association was found between neurological disorders and altered gene expression of ETNPPL, which decreased by 72% in the prefrontal cortex of post-mortem depressed patients [23,24]. Tsujioka and Yamashita [25] performed a study on the basal difference between neonatal and adult spinal cords and observed that *ETNPPL* gene levels were reduced in neonates and slightly suppressed after the surgical sectioning of one or both bulbar pyramids in adult mice, thus indicating that gene expression negatively correlated with axonal growth. Translationally, the authors concluded that ETNPPL might detect key aspects of neural development and repair of specific neural circuits.

Finally, according to the NCBI gene database, the ETNPPL protein is predominantly expressed in the brain (https://www.ncbi.nlm.nih.gov/gene/64850, accessed on 12 November 2024), possibly indicating a role in the neurodegenerative processes involved in PD and other degenerative disorders [26]. Shao and Vawter [27] identified an overexpressed *AGXT2L1* gene in the post-mortem brains of subjects with schizophrenia or bipolar disorder, compared to healthy controls; they hypothesized a functional dysregulation that might account for these diseases or represent part of a compensatory response to the neurochemical imbalance underlying these disorders.

Based on our previous findings, which this study aims to extend [20], and considering the complexity of PD and the relevant literature, we conducted an IHC analysis of SNc sections from PD patients and controls to evaluate the expression of the ETNPPL protein, which was significantly under-expressed in PD. Additionally, we assessed ETNPPL mRNA expression using Quantitative Real-Time PCR (qRT-PCR) on the same sample set.

## 2. Results

**Figure 1:**
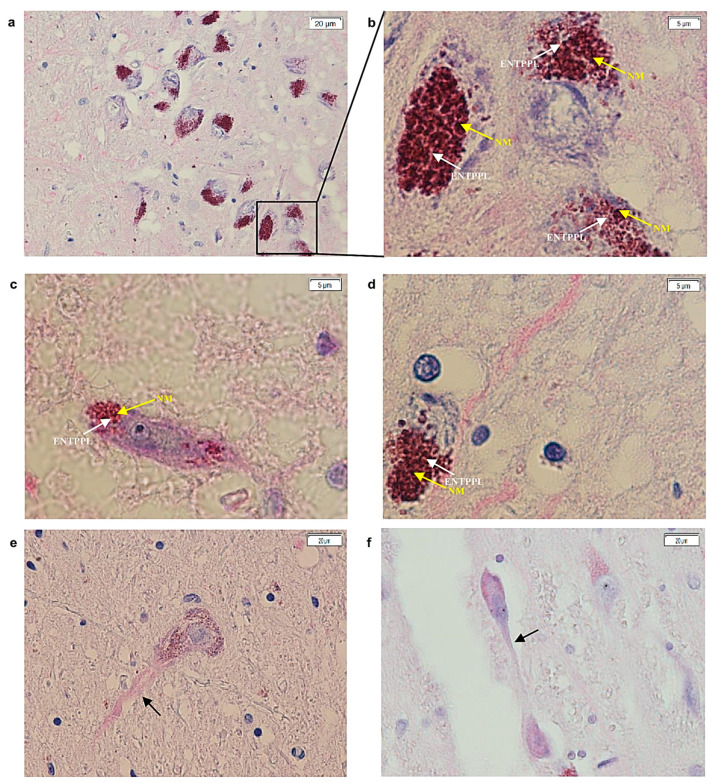
Images acquired with cellSens software (version 1.18) of sections of the substantia nigra from postmortem brains of healthy controls. (**a**–**d**) In particular, there is magenta staining (white arrow) given by the formation of the antigen–antibody complex at the cytoplasmic level, that recognizes the ENTPPL protein, as well as in the same areas where NM is present (yellow arrows). (**b**) is a magnification of (**a**), particularly the section within the square ((**a**), 20× and (**b**–**d**), 100×). (**e**,**f**) Magenta signal localized at the level of the neuronal axon (40×) (black arrowheads). ENTPPL: ethanolamine-phosphate-lyase; NM: neuromelanin; 20×, 100× and 40× is it is the magnification of the image with the lens.

ETNPPL protein expression was observed in all control samples. Specifically, the protein, identified by magenta staining, was, primarily, distributed in the area of the soma occupied by the dark neuromelanin (NM) granules of dopaminergic neurons in the SNc. The magenta color distinguished the protein from the typically brown appearance of NM (Figure 1). In these control samples, the protein was rarely found free in the cytoplasm and was also observed in some axons. No nuclear staining was detected.

**Figure 2:**
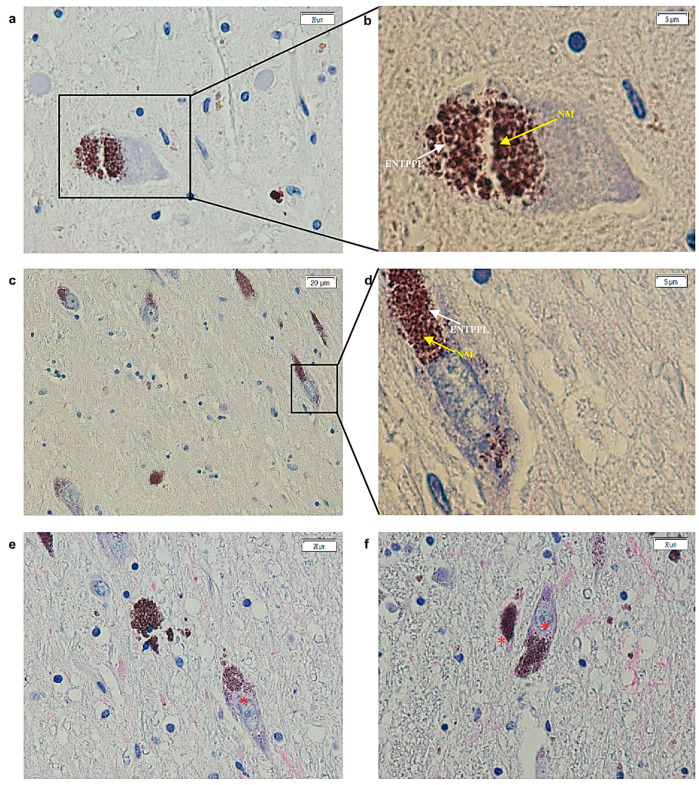
Images acquired with the cellSens software (version 1.18) of substantia nigra sections from post-mortem brains of PD subjects. (**a**–**d**) In all images, the lack of magenta signal (white arrows), which indicates the absence of binding to the ENTPPL protein, is evident in areas where NM was present, with typical brown staining (yellow arrows). (**b**,**d**) are magnifications of (**a**,**c**), respectively, as highlighted by the black box. ((**a**) 40×; (**b**) 100×; (**c**) 20× and (**d**) 100×). (**e**,**f**) Slight cytoplasmic and perinuclear signals can be observed in neurons (red asterisks) and not at the level of NM (40×). ENTPPL: ethanolamine-phosphate-lyase; NM: neuromelanin; 20×, 100× and 40× is it is the magnification of the image with the lens.

In contrast, the SNc dopaminergic neurons of PD subjects exhibited a weak cytoplasmic signal for the ETNPPL protein, with no nuclear or axonal staining observed (Figure 2). Additionally, unlike in the controls, the ETNPPL signal in PD samples was not associated with NM in the SNc dopaminergic neurons. No significant differences in the presence/absence of the magenta signal were found between the two groups either in relation to age or even gender.

**Figure 3:**
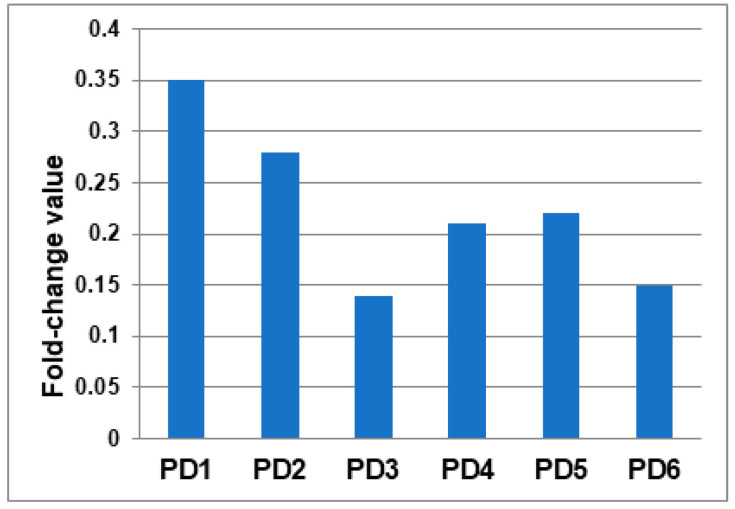
*ETNPPL* gene expression bar chart. Data obtained by qRT-PCR analysis on six human brain sections of PD subjects with a mixture of the six cDNAs of control subjects (the value for controls is always 1—not included in the table, control is mix cDNA of the six controls). Data shown were obtained by qRT-PCR by comparative ΔΔCt method; PD: Parkinson’s disease. ENTPPL: ethanolamine-phosphate-lyase; qRT-PCR: Quantitative real-time Polymerase Chain Reaction; PD: Parkinson’s Disease; cDNA: complementary DNA, DNA that is synthesized in vitro from a mature RNA template by the action of reverse transcriptase; ΔΔCt: analytical data from which the expression ratio or abundance of the target gene in the sample is obtained.

We found a reduction in ETNPPL mRNA levels in all six PD cases compared with the mRNA mixture of the six controls (Figure 3), which go hand in hand with the IHC outcomes obtained here, with a lack of signal in PD subjects compared with controls for ETNPPL protein at the level of SNc dopaminergic neurons but, also, with the results obtained in previous RNA sequencing data [20].

## 3. Discussion

NM is primarily found in the central nervous system, especially in primates. It is abundant in various brain regions, particularly in SNc and the locus coeruleus, the primary sources of dopaminergic and noradrenergic innervation, respectively.

In healthy individuals, NM is densely packed in the cytoplasm of these neurons and begins accumulating in early childhood. Structurally, NM is an insoluble, amorphous complex with a variable composition [16].

In 2021, Korzhevskii and colleagues [16] detailed the structural, cytochemical, and localization characteristics of NM in the SNc of neurologically healthy subjects across different ages. Their research focused on tyrosine (a precursor of dopamine and norepinephrine) and the enzyme tyrosinase, both involved in the synthesis of peripheral melanin (eumelanin and pheomelanin), though their roles in NM synthesis remain uncertain. Microscopic analysis of the SNc, where dopaminergic neurons are rich in NM granules, revealed immunoreactivity to tyrosine hydroxylase, a marker for dopaminergic neurons. This immunoreactivity decreased with age, leading to the hypothesis that declining tyrosine hydroxylase levels may indicate dysfunction in SNc dopaminergic neurons. Additionally, they proposed that NM accumulation could have harmful effects on these neurons, potentially playing a role in the etiology and pathogenesis of PD [16].

In the present study, we observed ETNPPL protein in the regions where NM is found in the SNc dopaminergic neurons of control subjects. However, in PD patients, this association was absent. Instead, the ETNPPL signal was dispersed within the cytoplasm of SNc neurons, with no nuclear localization. These findings were further validated through qRT-PCR analysis which also confirms the transcriptomic data recently described by Salemi et al. [20]. Therefore, both methods of mRNA expression detection correlated with the protein expression data themselves.

In contrast, another study observed ETNPPL protein in both the cytoplasm and nucleus of astrocytoma cells. Its expression was diffuse in low-grade tumors and diminished with malignancy, such as in high-grade gliomas [28]. The reduction in ETNPPL in high-grade gliomas suggests that this enzyme might counteract cell proliferation, which is otherwise heightened in such tumors. As previously mentioned, the *ETNPPL* gene is involved in the catabolism of phosphoethanolamine, a key compound in the synthesis of membrane phospholipids. Phosphatidylethanolamines, enriched in nerve tissue, along with ethanolamine and phosphoethanolamine, act as potent mitogens for cancer cell lines [29,30]. Therefore, the high expression of ETNPPL in low-grade gliomas and its under-expression in high-grade gliomas may indicate that reduced phosphoethanolamine and phosphatidylethanolamine synthesis might inhibit glioma cell growth [28].

Applying these findings to our study, ETNPPL expression in SNc neurons of healthy subjects may contribute to the physiological stability of these neurons. While nuclear localization of ETNPPL has been observed in gliomas and glioblastomas—particularly when over-expressed this does not correlate with the SNc neurons of either PD patients or controls. We hypothesize that the presence of ETNPPL in the cytoplasm, and occasionally in some axons, although scarcely detected in PD, may represent an inactive form of the protein.

The role of ETNPPL in neurodegenerative disorders, particularly PD, extends beyond its observed expression patterns. The significant decrease in ETNPPL protein expression in the substantia nigra of PD patients, compared to healthy controls, suggests its involvement in the pathological processes underlying dopaminergic neuron degeneration. This aligns with studies linking ETNPPL to phospholipid metabolism, a crucial factor in maintaining cellular membrane integrity and function [21,29,30]. Schiroli et al. [21] found that the *AGXT2L1* gene should be finely regulated, because its reaction is irreversible and generates toxic products. When assessing the mechanisms of gene action in neuropsychiatric diseases, it seems that its increased expression may help to mitigate cell membrane degradation processes; however, prolonged overregulation could lead cells to excessive exposure to acetaldehyde and ammonia, as well as to altered homeostasis of sphingolipids and glycerophospholipids. Consequently, we can assume that this gene may have pleiotropic effects and, therefore, implications at the brain level in different diseases. In our study, *AGXT2L1* was found to be under-expressed in PD subjects and this has been associated with the maintenance of cell membranes in the previous literature. Consequently, we can assume that, differently from the hypotheses by Schiroli et al. [21], the protein cannot mitigate the degradation of neuronal membranes or MN in PD subjects, at least at this stage of the disease. Disruption of these processes may contribute to the neurodegenerative cascade characteristic of PD, warranting further investigation into ETNPPL as a potential biomarker or therapeutic target.

The use of biomarkers in diagnostic and/or prognostic settings appears to be of increasing interest, especially for those diseases, such as neurodegenerative disorders, which represent a high healthcare and social burden. Although, to date, none has been validated for clinical practice, some candidates that appear to be promising are neuromelanin antibodies, pathological forms of α-synuclein, DJ-1 protein and gene expression, metabolomics, and protein profiling models [31,32]. For instance, Ding et al. [33] found that five serum miRNAs (miR-195, miR-185, miR-15b, miR-221 and miR-181a) can accurately distinguish PD patients from healthy individuals. Jang et al. [34] obtained differentially expressed genes (DEGs), from the Gene Expression Omnibus database, between MP groups and normal controls, and nine among the many dysregulated genes were indicated as useful biomarkers, e.g., prostaglandin D2 synthase (*PTGDS*), glutathione peroxidase 3 (*GPX3*), solute carrier family 25 member 20 (*SLC25A20*), calcium voltage-gated channel subunit alpha1 D (*CACNA1D*), leucine rich repeat neuronal 3 (*LRRN3*), RNA polymerase I and III subunit D (*POLR1D*), Rho GTPase activating protein 26 (*ARHGAP26*), TNF superfamily member 14 (*TNFSF14*), and VPS11 core subunit of CORVET and HOPS complexes (VPS11); subsequently, the authors examined their expression in blood samples of PD patients by qRT-PCR and concluded that these might serve as potential biomarkers of PD. ETNPPL protein expression can be compared with other known markers of neurodegeneration. Unlike α-synuclein, which forms pathological aggregates that drive disease progression [6,7], ETNPPL appears to be significantly under-expressed, suggesting a protective role that is compromised in PD. This contrast could provide new insights into the molecular mechanisms of neurodegeneration, highlighting the multifactorial nature of PD and the complex interplay of different pathological proteins.

The differential expression of ETNPPL in PD compared to healthy subjects presents potential avenues for novel therapeutic approaches. Targeting the pathways associated with ETNPPL, possibly through gene therapy or small-molecule modulators, could restore its function in dopaminergic neurons, potentially slowing or halting PD progression. Additionally, using ETNPPL as a biomarker for early diagnosis or for monitoring therapeutic efficacy could have a significant clinical impact. The current findings underscore the importance of integrating molecular insights into the development of targeted treatments for neurodegenerative diseases.

Limitations of this study are the sex imbalance between PD and controls, as well as the small sample size, mainly due to the difficulties in obtaining post-mortem brain tissue for analysis. Thus, this preliminary study focused on the evaluation of the protein synthesis product of a transcript identified as under-expressed from previous research [20] and related validation concerning the ETNPPL gene by qRT-PCR. However, all the PD subjects included were at Braak stage 6 and, given that no significant difference in the immunohistochemical signal was identified between males and females, we considered a possible sex-related difference unlikely. In any case, the histological sections available did not allow us to go into any possible sex differences potentially affecting the expression of the ETNPPL protein in this region of the human brain. Nevertheless, in future larger studies, we plan to investigate the translation of other differentially expressed genes found in the previous study and also to use Western blot, which could not be performed.

## 4. Materials and Methods

### 4.1. Post-Mortem Brain Sections

Human brain sections from subjects with PD and controls mounted on slides were obtained from the Multiple Sclerosis and Parkinson’s Tissue Bank of the Imperial College of London, Hammersmith Hospital Campus Du Cane Road (London W12 ONN, UK). Each section was 4 µm thick and was formalin-fixed paraffin-embedded (FFPE). A total of 6 PD samples (4 males and 2 females; mean age 79.50 ± 5.39 years) and 6 control samples (1 male and 5 females; mean age 78.17 ± 10.38) were acquired.

Table 1 shows the main demographic and clinical characteristics of all samples, with data made available from the abovementioned bank.

**Table 1:** Clinical characteristics of subjects analyzed by immunohistochemistry.

	Age	Sex	Braak LB Stage	Clinical Diagnosis
PD patients				
PD1	89	F	Parkinson’s/Lewy body findings, Braak LB stage 6, Limbic Lewy body disease	Parkinson’s disease
PD2	76	F	Parkinson’s/Lewy body findings, Braak LB stage 6, Amygdala-only Lewy body disease	Parkinson’s disease
PD3	79	M	Parkinson’s/Lewy body findings, Braak LB stage 6, Neocortical Lewy body disease	Parkinson’s disease
PD4	80	M	Parkinson’s/Lewy body findings, Braak LB stage 6, Neocortical Lewy body disease	Parkinson’s disease
PD5	73	M	Parkinson’s/Lewy body findings, Braak LB stage 6, Neocortical Lewy body disease	Parkinson’s disease
PD6	80	M	Parkinson’s/Lewy body findings, Braak LB stage 6, Neocortical Lewy body disease	Parkinson’s disease
Healthy controls				
Control1	87	F	-	No abnormality
Control2	59	F	-	No abnormality
Control3	74	F	-	No abnormality
Control4	84	M	-	No abnormality
Control5	84	F	-	No abnormality
Control6	81	F	-	No abnormality

Braak LB Stage: traditionally, PD develops across six neuropathological stages, which are called Braak’s Stages. Each stage is evidenced by typical inclusion bodies in the form of spindles or threads and, in part, as Lewy neurites (LNs), granular aggregations, pale spherical bodies, and/or Lewy bodies (LB), in different brain areas [35]; F: female, M: male; age expressed in years; PD: Parkinson’s Disease.

The study was carried out according to the principles of the 1964 Declaration of Helsinki and its following amendments. The Ethics Committee of the Oasi-IRCCS Research Institute of Troina (Italy) approved the protocol on 5 April 2022 (approval code: 2022/04/05/EC-IRCCS-OASI/52).

### 4.2. Immunohistochemistry (IHC)

To perform IHC analysis, tissue sections were first deparaffinized, a crucial step to fully expose the target antigen and facilitate the formation of the Ag-Ab complex. Since paraffin is hydrophobic and would repel aqueous staining solutions, the sections were then rehydrated to ensure proper interaction with the staining reagents [19,36]. Heat-induced epitope retrieval (HIER) was subsequently performed to unmask the antigen, which may have been altered during the fixation process, thereby restoring the tertiary structure needed for effective Ag-Ab binding [19]. This procedure followed the manufacturer’s guidelines using Coplin jars and the EnVision FLEX Mini Kit, High pH (Link)-K8023 (Agilent-Dako, Santa Clara, CA, USA).

For IHC, rabbit polyclonal anti-AGXT2L1/ETNPPL primary antibody (MBS9403063) was used at a 1:200 dilution (MyBioSource, Inc.; San Diego, CA, USA), diluted in EnVision FLEX Antibody Diluent (K8006), also provided by the EnVision FLEX Mini Kit. The tissue sections were incubated with the primary antibody for approximately two hours.

The EnVision kit also included EnVision FLEX Peroxidase-Blocking Reagent (SM801) (Agilent-Dako, Santa Clara, CA, USA), which was used to block endogenous peroxidase (HRP) activity, thereby reducing background staining [37]. This reagent was applied for three minutes according to the protocol. EnVision FLEX/HRP (SM802) (Agilent-Dako, Santa Clara, CA, USA), a buffered solution containing dextran coupled with multiple HRP molecules and goat antibodies against rabbit and mouse immunoglobulins [38], was applied for 20 min as per the protocol.

To visualize the Ag-Ab complex, a working solution was prepared by mixing 17 parts of EnVision FLEX HRP Magenta Substrate Chromogen System (DM857) (Agilent-Dako, Glostrup, DK) with three parts of EnVision FLEX Substrate Buffer (DM843) (Agilent-Dako, Glostrup, DK), following the manufacturer’s instructions (Agilent-Dako, Glostrup, DK). After the IHC reactions were completed, the sections were counterstained with hematoxylin. Control reactions recommended by the antibody manufacturer were performed for all IHC procedures. Finally, the sections were dehydrated and mounted using a xylene-based DPX mount (BDH, Pool, UK).

### 4.3. Microscopy

The IHC-processed sections were examined using an Olympus BX50 microscope (Olympus Italia S.r.l., Segrate, Italy) and photographed using an attached camera. Images were captured and analyzed using Olympus cellSens Standard software (version 1.18). Observations were made at magnifications of 20×, 40×, and 100× under oil immersion. The fraction of ETNPPL-positive cells was evaluated independently by two blinded coauthors (M.S. and F.A.S.), and no significant differences were found between the two observers.

### 4.4. RNA Isolation from Human Midbrain Samples and cDNA Production for qRT-PCR

RNA was extracted from 4 µm sections mounted on FFPE using the RecoverAll Total Nucleic Acid Isolation Protocol (Thermo Fisher Scientific Inc., Waltham, MA, USA), following the manufacturer’s instructions. Subsequently, the RNA was stored at −80 °C until further processing. Genomic DNA elimination reaction was performed using QuantiTect Reverse Transcription Kit (Qiagen, Hilden, Germany), with thermocycler program: 2′ min at 42 °C. Reverse transcription, required for cDNA synthesis from previously extracted RNA, was performed using 100 ng of RNA and QuantiTect Reverse Transcription Kit (Qiagen Sciences, Germantown, MD, USA), with thermocycler program: 15′ at 42 °C and 3′ at 95 °C.

### 4.5. Real-Time Quantitative PCR (qRT-PCR)

We performed qRT-PCR in the 6 FFPE sections of PD patients and created a control mixture of the 6 FFPE sections of control subjects. The qRT-PCR experiments were performed using LightCycler 480II (Roche Diagnostics; Mannheim, Germany) in a total volume of 25 μL. *ETNPPL* target gene assay (Hs00229818_m1) and glyceraldehyde-3-phosphate dehydrogenase (GAPDH) reference gene assay (Hs99999905_m1) were obtained from Applied Biosystems (Thermo Fisher Scientific Inc., Waltham, MA, USA). Thermocycling conditions consisted of a 2-min cycle at 50 °C (UDG incubation), a 15-min cycle at 95 °C (enzyme activation), and 42 cycles of 15 s at 94 °C followed by 1 min at 60 °C (PCR). The kit used was QuantiTect probe PCR Kit (Qiagen Sciences, Germantown, MD, USA). Amplified transcripts were quantified by the threshold cycle (Ct) method, and relative quantification analysis data were reproduced by the comparative ΔΔCt method: each cDNA from PD subjects was compared with a mixture of cDNAs from normal subjects. The LightCycler 480II SW 1.5.1.62 software provided with the LightCycler 480II was used for the relative quantification analysis.

## 5. Conclusions

IHC is a convenient analytical technique to assess the expression of proteins in cells of any tissue, including those obtained from subjects affected by neurodegenerative diseases. The data presented here on the protein expression of ETNPPL in SNc sections by IHC confirm the mRNA sequencing results of our previous study [20], in which its transcript was strongly underexpressed (PD vs. CTRL) [20], and, these results, align with the qRT-PCR results in this study. We then extended the results of previous work [20] by localizing a higher presence of the ETNPPL protein at the NM level in the SNc neurons of control subjects. To the best of our knowledge, this is the first report of reduced ETNPPL expression in PD patients and, therefore, the lack of previous evidence prevents a more comprehensive overview of the available studies and a deeper interpretation of the result. We cannot exclude that ETNPPL may play a protective role on dopaminergic neurons closely related to neuromelanin. Since this function is lacking, we hypothesize that it may somehow take part in the pathomechanisms of the affected individuals and that this protein could tend to localize in other locations of the neuron itself.

Despite the small sample size and the preliminary nature of the results, which prevents us from drawing definitive conclusions, this study lays the foundation for future research, first focusing on the impact of the ETNPPL gene in neurodegeneration and, at a later stage, on its possible role for diagnosis and prognosis; indeed, after additional research, it may be proposed as a potential diagnostic biomarker or new therapeutic target.

## Data Availability

Due to privacy restrictions, data are available from the senior author upon reasonable request.
